# Grandparents’ and Grandchildren’s Shared Festive Leisure

**DOI:** 10.3390/ijerph18168850

**Published:** 2021-08-22

**Authors:** María Ángeles Valdemoros San Emeterio, Ana Ponce de León Elizondo, Rosa Ana Alonso Ruiz, Magdalena Sáenz de Jubera Ocón, Eva Sanz Arazuri

**Affiliations:** Department of Ciencias de la Educación, University of La Rioja, 26004 Logroño, Spain; ana.ponce@unirioja.es (A.P.d.L.E.); rosa-ana.alonso@unirioja.es (R.A.A.R.); m-magdalena.saenz-de-jubera@unirioja.es (M.S.d.J.O.); eva.sanz@unirioja.es (E.S.A.)

**Keywords:** well-being, festive leisure, grandparents, grandchildren

## Abstract

Festive leisure provides experiences that can generate intergenerational well-being. The study aimed to examine the festive leisure activities shared by grandparents and grandchildren, and the link with times, spaces, motives, and well-being that these activities bring to both generations. A cross-sectional telematic survey was carried out with 357 grandparents living in the northern part of Spain, who had grandchildren aged between 6 and 12 years. Both a descriptive and inferential analysis was performed. A high proportion of grandparents and grandchildren share festive activities, which occur on weekends and holiday periods. Private spaces, such as bars, cafeterias, and restaurants are the ones chosen for going out to eat or drink, and open public spaces like parks, squares, and streets are dedicated to traditional festivals, and are excellent scenarios for coexistence and intergenerational social interaction. The reasons that drive this practice are associated with the strengthening of emotional ties and family intimacy. Grandparents consider the practice of shared festive leisure to be beneficial for their personal development because they perceive that, thanks to this leisure, they improve their creativity, physical condition, their happiness and fun, the relationship with their grandchildren, and develop new manual and technical skills.

## 1. Introduction

Leisure, as a universal right, generates personal growth and enhances the bonds among the people who experience it, providing free, positive, and valuable experiences [1,2,3]. A complex phenomenon, it is a reality that differs in its expression, contexts, and dimensions, such as recreational, creative, festive, environmental-ecological, and solidary [3,4].

Specifically, the festive dimension is a social manifestation of leisure aimed at celebrating some event linked to a joyful, happy atmosphere of freedom and free consent [5]. Festive leisure activities are the ones most commonly carried out with the family, taking the form of experiences such as festivals and celebrations, as well as going out to eat or drink in a restaurant or a bar [6]. 

The nature of collective festive leisure with recreational and liberating functions involves a social and cultural approach, or a more personal and familiar approach, manifested in events such as traditional festivals and celebrations, oral traditions, performing arts, social uses, rituals, beliefs and knowledge, as well as techniques linked to traditional craftsmanship. They are considered living practices and expressions inherited from ancestors and transmitted to descendants and are included in one’s intangible cultural heritage or living heritage [2].

In 2003, in its Article 20, the UNESCO Convention for the Safeguarding of the Intangible Cultural Heritage listed festive leisure among the objectives of the intangible cultural heritage that require urgent measures (https://n9.cl/2bkn accessed on 15 June 2021). 

Prior to the home confinement and mobility restrictions established to control the spread of COVID-19, some studies [5,6,7] found that festive celebrations (commemorations, birthdays, anniversaries, etc.) were a very relevant aspect of people’s lives, considering that they facilitated meetings, relationships with others, relaxation, and enjoyment or happiness. Sometimes these celebrations relegated their transcendental and extraordinary nature, often linked to religious aspects, to enhance their secular nature, making them a part of everyday life. The festivals took place in leisure spaces, a faithful reflection of society’s culture and values, and were part of the ancestors’ cultural legacy.

Festivals are experienced differently depending on the related factor of people’s age. It has been found that, as people age, they prioritize the relationship with loved ones in celebrations [5]. 

The health situation of the global pandemic due to COVID-19 hindered the physical contact of grandparents and grandchildren [7,8,9], a circumstance that shows the need to contribute with research that will once again promote intergenerational festive exchanges, and resume the impulse to acknowledge the grandparents as bearers of an extensive cultural background, although safely and healthily.

Older people have more leisure time [10] and they play an active role in participating in and contributing to the dissemination of traditions, customs, and intangible cultural heritage, especially in their relationships with their grandchildren. This contributes to a collective memory that makes use of our ancestors’ legacy of customs and traditions to offer them to future generations in a context of family affection, which is a frame of reference for social construction [11]. Festive leisure connects the two generations and fosters intergenerational feelings of belonging to these experiences with which they identify [12,13]. 

Grandparents’ decisions to transmit traditional knowledge and values are usually active choices driven by the wish to leave their descendants a legacy of traditions that ensures cultural continuity in their grandchildren as the bearers of social and cultural capital [14,15]. In studies such as those of [16,17,18,19,20,21], the authors highlight that grandmothers are more active mediators in intergenerational cultural exchanges than grandfathers, although there are no significant differences between them in terms of the degree of participation in the socialization of their grandchildren [22].

This vision of sharing festive leisure between grandparents and grandchildren through exchanges, contacts, and memories, etc., addresses areas of socialization, formation, and transmission of historical knowledge, tradition, culture, and values, as well as identity and membership in a community [14,15,16,17,18]. With the passing of time, these intergenerational relationships, which are initially asymmetrical and nonreciprocal in the transmission of knowledge, help children to participate and, little by little, to appropriate the world, in addition to maintaining their cultural heritage, roots, and identity. The grandchildren may even inspire their grandparents to learn about current traditions and customs [13].

In short, the experiences shared by grandparents and grandchildren promote knowledge, exchange, and mutual learning. They strengthen relationship ties, increase and strengthen bonds through positive feedback, and provide psychological, social, educational, and cultural benefits, in addition to turning these joint experiences into a source of human development for both generations [12,23,24,25,26,27,28].

It is important to enjoy leisure experiences in intergenerational contexts from an early age. The present study focuses on the ages that make up the stage of primary education (6–12 years), as previous studies have found that, at these ages, grandparents are generally highly involved in the care of their grandchildren [29] and they consider that the positive effects of these experiences will be consolidated in subsequent life stages [30,31].

The objective of this research is to examine the festive leisure activities shared by grandparents and grandchildren, and the link with the times and spaces in which these are shared, the reasons that guide them, and the well-being they provide to both generations.

The state of the issue leads us to hypothesize that festive leisure is one of the leisure activities most shared by grandparents and grandchildren. As festive leisure implies a break in work and student obligations, the weekends and holidays are probably the preferred periods for the practice of festive leisure shared by grandparents and grandchildren (Hypothesis 1). In addition, we hypothesize that the spaces used the most will be associated with the type of shared festive leisure (Hypothesis 2).

## 2. Materials and Methods

### 2.1. Study Design

A cross-sectional telematic survey was carried out with grandparents living in the northern part of Spain, who had grandchildren between 6 and 12 years of age.

The researchers contacted the participants by telephone.

### 2.2. Participants

This work is part of a larger study that analyzes, in the north of Spain, grandparents’ and grandchildren’s shared leisure, shared activities, frequency, where they are shared, the activities they would like to share, and their perceived satisfaction with shared leisure. The study population was made up of the total number of 6 to 12-year-old children, living in the north of Spain, in addition to their living grandparents. Taking into consideration that, in the Spanish state, every child from 6 to 12 years of age has to be enrolled in an educational center and that the population of grandparents with grandchildren between 6 and 12 years old was difficult to identify, the study population was defined from the statistical data published by the ministries and education departments of each of the autonomous communities that make up the northern area of Spain. The data collected showed a population size of 250,357 primary education students, living in northern Spain, during the academic year 2019/2020.

Setting an absolute error of 3%, a 95% confidence level, and considering the assumption of p = q = 0.5, the sample size was estimated at 1075 students. With an experimental mortality of 1.11%, the final sample size consisted of 1063 students.

The final sample units of students were selected using proportional sampling, stratified by province and clusters, selecting educational centers in each of the provinces, and interviewing all the students from the classrooms chosen from among the randomly selected centers. To be part of the sample, it was an essential requirement for each student to have the informed and signed consent of their parent or legal guardian. In the authorization form, the parents or legal guardians were asked to provide the phone number of a grandparent of the child. We obtained 357 phone numbers of grandparents.

**Figure 1:**
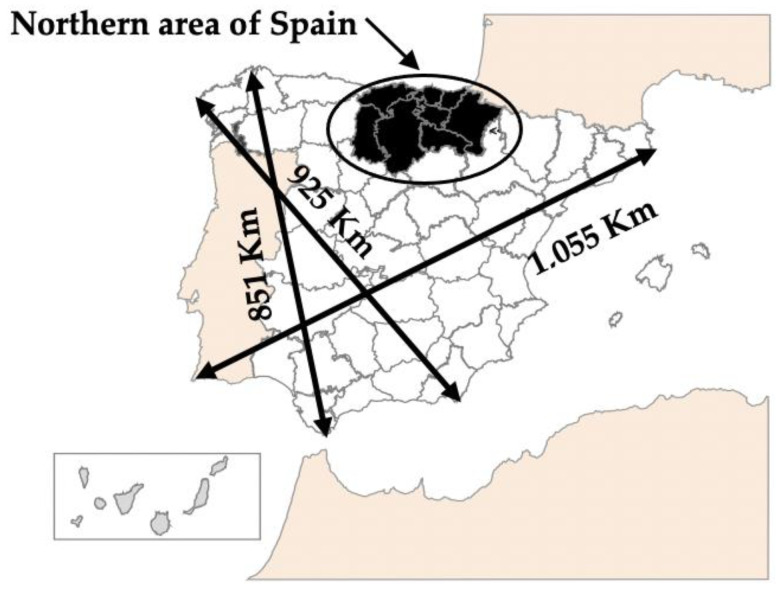
Northern area of Spain: Cantabria, Biscay, Gipuzkoa, Alava, La Rioja, Navarre, Burgos, and Palencia (shaded in black).

The 357 grandparents whose telephone numbers we obtained made up the specific simple of the work presented in this article. They lived in the northern part of Spain, made up of 8 Spanish provinces: Cantabria, Biscay, Gipuzkoa, Alava, La Rioja, Navarre, Burgos, and Palencia (Figure 1). These 8 provinces have a population of 250,357 primary school students between the ages of 6 and 12, according to statistical data published by the ministries and departments of education of each autonomous community. Of the participants, 25.3% of the grandparents were male and 74.7% were female. Concerning age, 25.2% of the grandparents were under the age of 65, while 51.8% were between ages 65 and 74, and 21.6% were 75 years old or older.

The selection of participants was carried out through the requests for permission and telephone numbers to the parents of a sample of 1075 students of primary education through stratified and proportional probabilistic selection by province (Table 1). The “Another” section shows that 5.6% of grandparents resided in a different province from their grandchildren, who lived in one of the provinces under study.

### 2.3. Instruments

The absence of a valid and reliable instrument to collect the relevant information for this study led to the development of an ad-hoc questionnaire after the review of the scientific literature to gather the data through 5 variables: space used to share leisure activities by grandparents and grandchildren, leisure activity shared by grandparents and grandchildren, geographical location of the place of residence of the grandparents, type of municipality where the grandparents live, and province of residence of the grandparents.

The variables recorded for this study are defined below:

- Shared leisure, a dichotomous variable that recorded whether any type of leisure activity is shared with the grandchildren; it could be cultural, recreational, festive, digital, environmental-ecological, and solidary. Through the question “Do you share any activity [each of the indicated dimensions is specified] with your grandchild(ren) aged between 6 and 12 years?”, the options established were: yes /no. If the participant responded affirmatively to the item of festive leisure shared with their grandchildren, they had to indicate which specific festive activities they shared. “Go out to eat or drink at a restaurant or a bar” is an activity with a strong festive component among Spaniards and therefore is identified among festive activities.
- Periodicity with which leisure is shared, a categorical variable that collected the sections of leisure time shared with the grandchild(ren), through the item “Indicate the frequency with which you perform each of the indicated activities” with 8 options: 1 = I do not share leisure with my grandchild(ren); 2 = only on vacation, 3 = only 1 or 2 days a month; 4 = on weekends, one day; 5 = on weekends, both days; 6 = on weekdays, 1 or 2 days; 7 = on weekdays, 3 or 4 days; 8 = on weekdays, all 5 days.
- Space used to share festive leisure activities between grandparents and grandchildren, a categorical variable that recorded the place where grandparents share some type of leisure activity with their grandchildren, through the question “Indicate the place where you perform each of the indicated activities”, with 8 options: 1 = at home (grandparents or grandchildren); 2 = in associations, societies, or clubs (associations are groups of persons constituted to carry out a collective activity in a stable manner, which have a registered office); 3 = in municipal spaces (sports centers, cultural centers, playrooms, etc.); 4 = in open public spaces in a town (street, park, square, etc.); 5 = in nature, natural spaces that are outside of the town; 6 = in school facilities.
- The reasons that lead the grandparent to share leisure with their grandchildren, a categorical variable that collected the reasons that guide the practice, through the question “Indicate the reasons why you practice festive leisure”, with 8 options: 1 = simply because I like it; 2 = I take care of them while their parents work; 3 = I have no other people with whom to share that activity; 4 = my grandchild(ren) have no other people with whom to share that activity; 5 = my grandchild(ren) know a lot about this activity and they teach me; 6 = I master this activity and teach my grandchild(ren); 7 = to entertain my grandchild(ren); 8 = to spend more time with my grandchild(ren).
- Benefits provided by leisure shared with the grandchild(ren), 5 categorical variables (physical benefit, emotional well-being, creative benefit, manual benefit, and social benefit) collected information on the contributions of festive leisure to the grandparents’ well-being, through five items: 1 = The leisure I share with my grandchild(ren) helps me to be fit, to control my movements, maintain or improve my physical condition; 2 = The leisure time I share with my grandchild(ren) helps me to be happier, enjoy that leisure more, and have more fun; 3 = The leisure I share with my grandchild(ren) helps me to be more creative; 4 = The leisure I share with my grandchild(ren) helps me develop new manual skills or perfect technical skills; 5 = The leisure I share with my grandchild(ren) helps me to relate better to them. This information is collected through 5 options (1 = strongly disagree; 5 = strongly agree).

### 2.4. Procedure

A cross-sectional telematic survey was carried out with grandparents who had grandchildren between 6 and 12 years of age and lived in the northern part of Spain. The questionnaire was applied to the grandparents of students from the different randomly selected schools in each of the 8 provinces that make up the northern part of the Spanish state. Before applying the instrument to the grandchildren, the consent of the parents or legal guardians of the children was requested, and the parents were asked to provide the private telephone number of a grandfather or grandmother of their children. Seven previously trained researchers personally called each participant by phone. Before starting each interview, participants were assured about the confidentiality of their responses, as well as the protection of their rights and guarantees. The answers were recorded by the researchers on the digitized questionnaire while they conducted the interview. The Ethics Committee of the university to which the researchers belong approved this procedure on December 17, 2019. The positive report of this Ethics Committee was recorded with the code CE_02_2019.

### 2.5. Analysis

A data analysis was performed in two phases by means of the SPSS 23.0 statistical program (IBM SPSS Statistics for Windows, Version 23.0. Armonk, NY, USA: IBM Corp).

Firstly, a descriptive study was carried out that revealed through frequency statistics the leisure activities that the grandparents generally shared with their grandchildren aged 6 to 12, in particular the typology of festive leisure activities that they carried out, as well as their periodicity, the places where they practiced them, and the motivations that guided their performance.

In the second phase, an inferential analysis was carried out through the Cochran and Chi-squared Q-tests, examining: (a) the presence of significant differences in the frequency of festive leisure practice; (b) the existence of significant differences in terms of the spaces where festive leisure is practiced; (c) the existence of significant differences in terms of the motivations that lead to the practice of festive leisure; and (d) the degree of association between the benefits produced by the shared practice and the experience of festive leisure.

The level of significance established for this study was set at *p* < 0.05. 

## 3. Results

**Figure 2:**
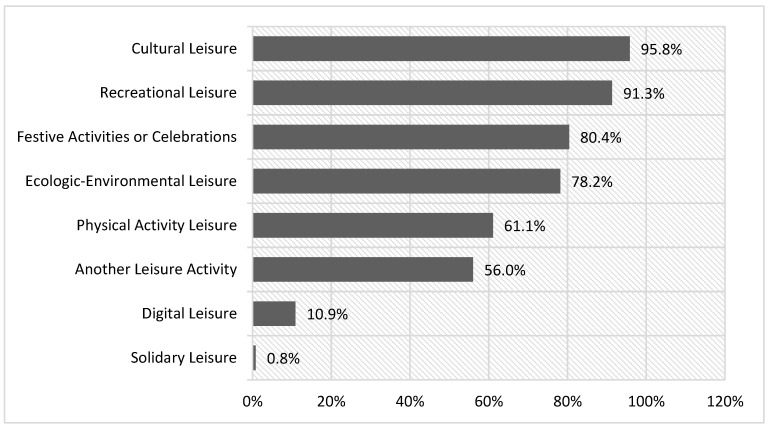
Typology of leisure activities practiced.

Festive leisure shared by grandparents and grandchildren is very widespread and is practiced by 80.4% (Figure 2). This percentage is lower than that of the majority activities, such as cultural leisure (95.8%) or recreational activities, but the difference between the percentages is low. However, it is higher than that of leisure physical activities (56%), with a greater difference, and well above the percentage of digital leisure (10.9%).

**Figure 3:**
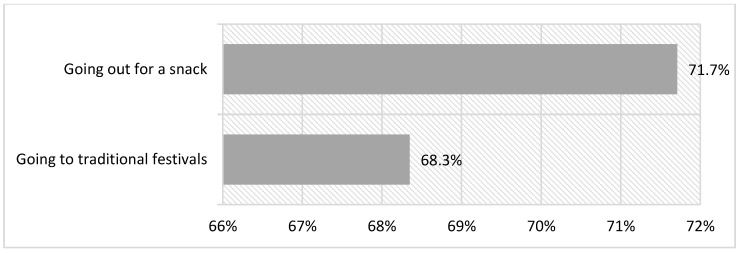
Festive leisure activities practiced.

Within the activities included in this typology, after carrying out a classification of the activities based on the scientific literature [32], we found no great differences between “Going out to eat or drink” and “Going to traditional festivals”, with the two experiences included in about 70% of the practice (Figure 3). Thus, “Going out to eat or drink” has a 3.4-point higher percentage than “Going to traditional festivals”; a fairly small difference in relative terms.

**Figure 4:**
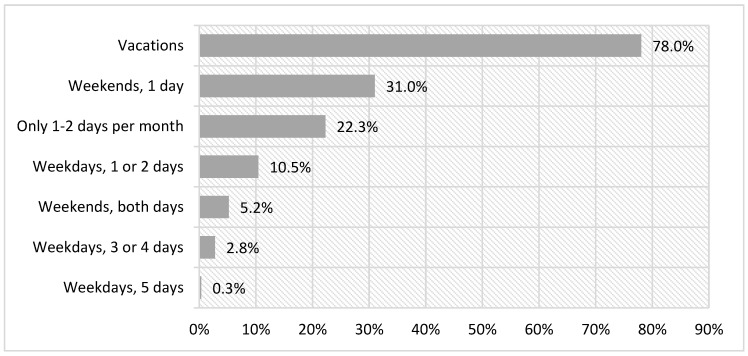
Frequency of shared practice of festive leisure.

As for the frequency of practice, there were significant differences related to the holiday periods and weekends (χ²(6) = 687.78, *p* < 0.000). The frequency of festive leisure that is most repeated is once on the weekend and once or twice on weekdays (Figure 4).

There are also significant differences between the two activities included in the category of festive leisure: “Going out to eat or drink” (χ²(6) = 1103.78, *p* < 0.000) and “Going to traditional festivals” (χ²(6) = 187.20, *p* < 0.000). The activity of “Going out to eat or drink” is practiced more frequently than “Going to traditional festivals”.

**Figure 5:**
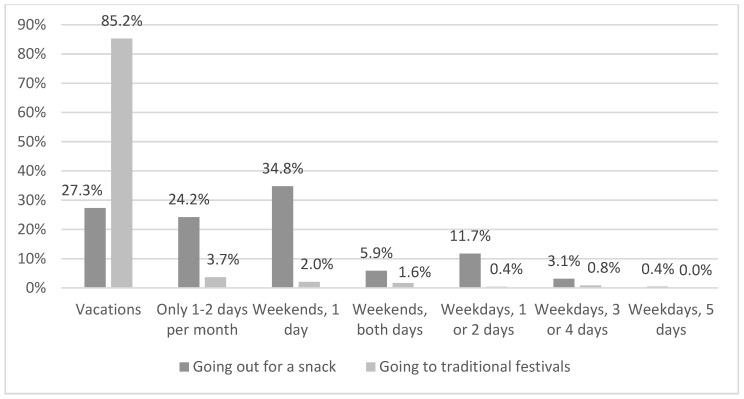
Frequency of shared festive leisure activities.

The percentages of the activity of “Going out to eat or drink” are less variable. It is practiced mostly on weekends, usually 1 day, to a lesser extent on vacations, and its practice during the week is the least common. As for traditional festivals, they are strongly linked to holiday periods, and practiced by 85.2% (Figure 5).

**Figure 6:**
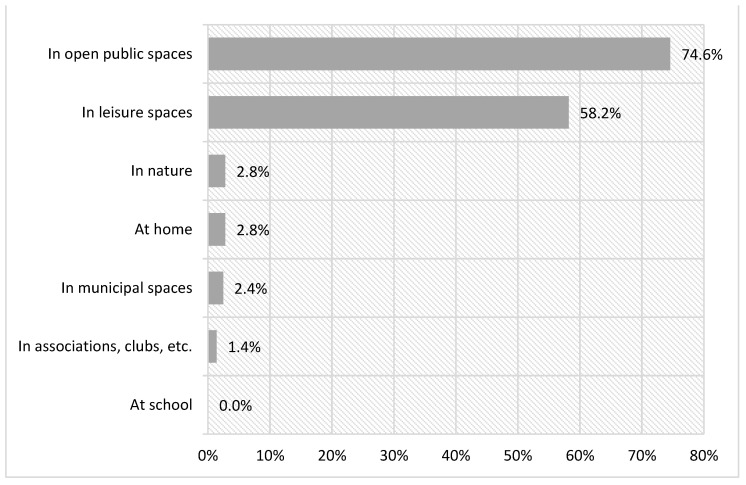
Places to practice festive leisure.

The practice of shared festive leisure takes place in two main locations: open public spaces and private leisure spaces, with statistically significant differences (χ²(6) = 377.73, *p* < 0.000). To examine this disparity in more detail, we can observe the places according to the specific activity carried out (Figure 6).

**Figure 7:**
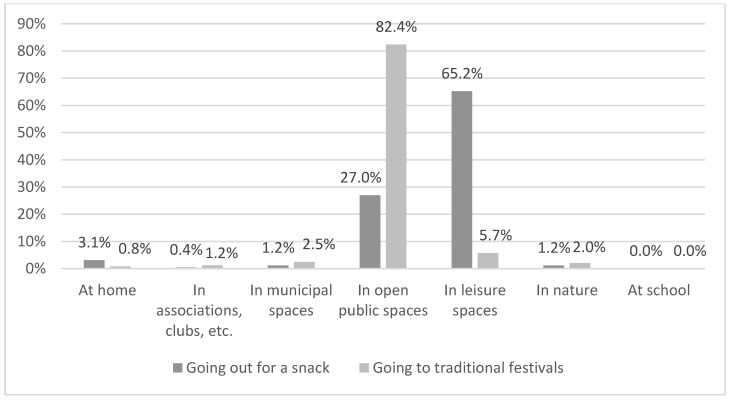
Places to practice festive leisure activities.

Going out to eat or drink is especially practiced (65.2%) in private leisure spaces. This includes places such as bars, cafeterias, restaurants, or shopping malls, the usual locations for this type of practice. To a lesser extent (27%), it is practiced in open public places, such as squares, parks, or streets. However, people mostly go to these places for traditional festivals (82.4%) (Figure 7).

The other locations indicated, such as at home, in associations, clubs, or municipal spaces are very unusual for the practice of festive leisure, according to the people surveyed.

**Figure 8:**
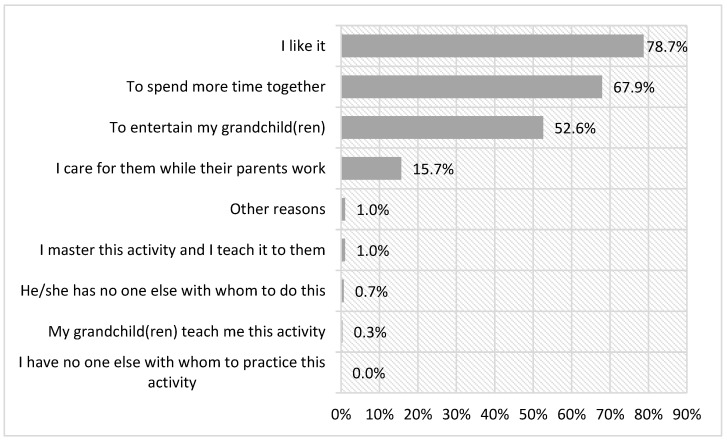
Reasons that guide the joint practice of festive leisure.

As for the motivations that lead this population to share festive leisure activities with their grandchildren, the most cited reasons are “because I like it” (78.7%) and “to spend more time together” (67.9%). At a lower percentage, but still cited by 52.6% of the surveyed population, the option “to entertain my grandchild” also appears. The option of care appears as the last relevant motivation with only 15.7%. The other motivations present a residual percentage (Figure 8).

When disaggregating the activities, significant differences were only found in the case of caring for the grandchild(ren) while the parents work (χ²(8) = 1299.84, *p* < 0.000), where the activity of going out to eat or drink presented a percentage of 15.2%, with 6.6 points of difference compared to going to traditional festivals. In the rest of the reasons, the pattern is repeated, with the motivations “I like it” and “to spend more time together” being the options with the majority (Table 2).

Festive leisure activities are associated with a higher level of benefits provided by shared leisure. The five parameters analyzed in this study reveal statistically different distributions for the groups that perform shared festive leisure activities, whose participants report greater benefits (Table 3).

The aid to creativity is the benefit with the clearest differences between the group that shares festive leisure and the group that does not share (Table 3). Among the people who do share festive leisure, 78.4% strongly agree with its effects on creativity, whereas, among those who do not share it, only 45.7% strongly agree.

The development of new manual and/or technical skills is perceived as a benefit among the population that shares this type of activity. There is more unanimous agreement in this group of people, with a percentage of 78.7% that strongly agrees with this statement, whereas only 54.3% of those who do not share festive leisure activities strongly agree that their leisure helps them to develop these types of skills. The percentage of negative responses to this statement is almost nine points higher among people who do not share festive leisure.

Festive leisure activities are also related to physical form. The differences in the percentages of negative responses are not as marked as in manual and/or technical skills; however, there is still a significant difference in terms of the maximum level of agreement, with a difference of 23 percentage points.

The association with the other two benefits analyzed—“it helps me to feel happier” and “it helps me to relate better to my grandchildren”—is not as strong as the association with the other benefits analyzed so far. However, people who share festive leisure also report to a greater extent the beneficial effect it has on them, with higher percentages in the “strongly agree” category for both benefits (Table 4).

## 4. Discussion

This study focused on analyzing the festive leisure activities shared by grandparents and grandchildren living in the northern part of Spain, and their link with the times and spaces of practice, the reasons that guide them, and the well-being they provide to both generations The study reveals that a high percentage of grandparents shared festive leisure activities with their grandchildren aged 6 and 12 years before the COVID-19 pandemic, and this percentage was only exceeded by cultural and recreational practices. Likewise, the two activities that are included within this type of leisure, “Going out to eat or drink” and “Going to traditional festivals”, are experiences shared by a large majority of grandparents and grandchildren. These results are in line with the findings obtained by the study of [5], in which the authors showed a remarkable assiduity of Spaniards for celebrations and attendance at festivals, which confirms that there is still an important tradition of festivities in leisure time.

This work also reveals the link between the kind of festive leisure experience and the period of its practice, which relates the shared practice with holidays and weekends. It is more common to go out to eat or drink on weekends and more typical of the holidays to go to traditional festivals. These differences can be understood due to the nature of these practices, because traditional festivals are usually celebrated in the summer in the Spanish context. On the other hand, going out to eat or drink has much fewer requirements, and can be done in less time.

In addition, the study proves that the typology of festive leisure conditions the spaces for its practice. Private spaces are reserved to eat or drink, and open public spaces are reserved for traditional festivals. These results were expected because these types of festivities are usually celebrated in these locations [15]. Research shows that the practice of these leisure experiences in public spaces has a positive impact on the participation and improvement of interpersonal relationships, as these spaces are ideal contexts for meetings, coexistence, and social interaction between different generations [33]. Grandparents and grandchildren do not use spaces such as the home, associations, or municipal spaces very frequently to carry out this type of experience.

Another relevant finding of this research is that intergenerational festive leisure activities are perceived by the grandparents as beneficial for their personal development because, as they are related to a happy, cheerful, and free environment, they are experienced as authentic leisure [2]. In addition, we underline the reasons concerning emotional bonds, which contribute to the establishment of stronger ties between grandparents and grandchildren and strengthen their complicity and alliance in the family environment [34]. Both reasons are in line with those of other experiences linked to the recreational dimension of leisure, where motivations associated with emotions and enjoyment predominate [5].

On the other hand, the grandparents do not agree with the contributions of experiences of festive leisure to intergenerational learning. They consider that practicing this type of leisure does not cover the need to teach and be taught in both parties. This is contrary to other types of leisure such as recreational and cultural leisure [35] and environmental-ecological leisure [34], in which the grandchildren instruct their grandparents in the performance of leisure practices that they master and the grandparents guide them in the performance of leisure activities about which they are more knowledgeable and have more experience. Leisure spaces are perceived by grandparents as scenarios where sharing leisure turns them into transmitters of historical knowledge, tradition, culture, and values, as well as of identity and membership in a community [9,20,21,22,36].

The differences in the types of festive leisure in terms of the reasons for its practice confirm that caring for the grandchildren while the parents work is significantly associated with going out to eat or drink with the children. This may be because this habit is rooted in the daily family life of many Spaniards.

The scientific literature confirms that sharing festive leisure activities provides numerous benefits to both generations, as it favors co-learning, strengthens affective bonds, and is a source of human development for both generations [12,13,24,25,26,31]. This study also shows that intergenerational festive leisure experiences are associated with high levels of benefits, specifically, sharing festive leisure implies a different perception of the benefits in creativity, physical form, enjoyment, manual skills, and the relationship between grandparents and grandchildren. Sharing this type of leisure increases the grandparents’ perception of the effect of these practices on the improvement of their creativity, the maintenance or increase of their physical condition, the contribution of happiness and fun, the consolidation of intergenerational relationships, the development of new manual skills, and the acquisition or improvement of technical skills.

Taking into account that festive leisure does not generally have a physical component, it is likely that this improvement in physical form is due to the fact that those who share festive leisure also share other types of leisure.

This article focuses on the festive leisure activities shared by grandparents and grandchildren and their link with the times and spaces of practice, the reasons that guide them, and the well-being that they provide to both generations. Intergenerational relations depend on a complex ecosystem of variables (age, gender, maternal or paternal grandparent, professional situation, the predominant role of grandparents with grandchildren, etc.) that cannot be considered in this case, but that are contemplated in a broader investigation within which this publication is framed.

## 5. Conclusions

A high proportion of grandparents and grandchildren share festive leisure activities, which materialize in going out to eat or drink and going to traditional festivals, and which are associated with weekends and holidays, respectively.

Private spaces, such as bars, cafeterias, and restaurants are chosen for going out to eat or drink, and open public spaces like parks, squares, and streets are dedicated to traditional festivals and are excellent scenarios for coexistence and intergenerational social interaction.

The reasons that drive this practice are linked to the strengthening of emotional ties and family intimacy.

The practice of shared festive leisure is considered by grandparents to be beneficial for their personal development, perceiving that, thanks to such leisure, they improve their creativity, maintain or increase their physical condition, promote their happiness and fun, consolidate the relationship with their grandchildren, develop new manual skills, and acquire or perfect technical skills.

### Strengths and Limitations

The results of this study are limited to the group of grandparents, so in future work the sample under study will increase through the participation of the grandchildren, and a more specific type of data analysis will be carried out to deepen our knowledge of the phenomenon investigated and contribute greater richness to the study.

As a limitation, social desirability may have increased the number of reflections on well-being perceived by grandparents and grandchildren, as previous studies verify that people tend to increase the degree of satisfaction and happiness in their responses when there are affective and emotional ties [37].

On the other hand, the timeline in which the research is framed highlights the reality of intergenerational leisure time before the COVID-19 pandemic. The prevention measures established to mitigate the spread of the virus, the suspension of festive events, and the limitations in the hotel industry, together with the fact that older people are considered especially vulnerable, have produced important alterations in the grandparent–grandchild relationship that also cause substantial changes in festive leisure practices. This situation shows the need to expand this research to understand the implications of the pandemic in intergenerational leisure, which leads to the rethinking of new dynamics that favor the practice of shared leisure activities to promote intergenerational well-being.

This study is based on information collected through interviews with participants. It could be expanded in the future with an experimental investigation with a control group and an experimental group.

Nonetheless, a notable strength of this study is the evidence of high participation in festive activities shared by grandparents and grandchildren and their associated benefits in the areas of creativity, physical condition, the contribution of joy and fun, as well as the consolidation of the relationship. Even in the times of the pandemic, festive activities stand out as an excellent tool of intergenerational leisure because, as they can be performed mostly outdoors, the incidence and possibility of contagion are much lower, which means that this shared leisure is carried out in safe contexts.

## Figures and Tables

**Table 1:** Target sample of the study. Grandparents of children in primary education (6–12 years) in northern Spain.

	Provinces	Frequency	Valid Percentage
NORTHERN SPAIN	Cantabria	35	9.9%
	Biscay	65	18.3%
	Gipuzkoa	19	4.9%
	Alava	46	13.0%
	La Rioja	90	25.3%
	Navarre	45	12.6%
	Burgos	15	4.2%
	Palencia	22	6.2%
	Another province	20	5.6%
	TOTAL	357	100%

**Table 2:** Motivations to perform the types of festive leisure activities.

Motivations	Going Out to Eat or Drink	Going to Traditional Festivals
I like it	75.8%	74.6%
To spend more time together	65.6%	61.5%
To entertain him/her	47.3%	52.0%
I take care of them while their parents work	15.2%	8.6%
Other reasons	0.8%	0.4%
He/she has no one else with whom to share it	0.4%	0.8%
I master this activity and I teach it to them	0.0%	1.2%
My grandchild teaches me this activity	0.0%	0.4%
I have no one else with whom to share it	0.0%	0.0%

**Table 3:** Chi-square test: benefits of festive leisure activities in grandparents who share compared with grandparents who do not share.

BENEFITS	χ^2^	df	*p*
The leisure I share with my grandchild(ren) helps me…			
…to be fit, to better control my movements, maintain or improve my physical condition (I am stronger, I run more…)	21.403	4	0.000 *
…to be happier, I enjoy doing it, it amuses me	10.661	4	0.031 *
…to be more creative	35.919	4	0.000 *
… to develop new manual skills and I acquire or perfect technical skills…	27.406	4	0.000 *
…to relate better to them	11.099	4	0.025 *

* *p* < 0.05.

**Table 4:** Benefits for grandparents who share festive leisure with their grandchildren versus grandparents who do not.

The Leisure I Share with My Grandchild(ren) Helps Me…						
		Strongly Disagree	2	3	4	Strongly Agree
…stay fit	Does not practice Festive Leisure	4.3%	4.3%	7.1%	25.7%	58.6%
	Practices Festive Leisure	1.4%	2.8%	5.6%	8.4%	81.9%
… to feel happier	Does not practice Festive Leisure	1.4%	1.4%	1.4%	10.0%	85.7%
	Practices Festive Leisure	0.0%	0.0%	0.7%	5.6%	93.7%
… be more creative	Does not practice Festive Leisure	2.9%	11.4%	18.6%	21.4%	45.7%
	Practices Festive Leisure	0.0%	4.2%	9.8%	7.7%	78.4%
… develop new manual and/or technical skills	Does not practice Festive Leisure	5.7%	7.1%	5.7%	27.1%	54.3%
	Practices Festive Leisure	0.3%	3.8%	5.9%	11.2%	78.7%
… relate better to my grandchild(ren)	Does not practice Festive Leisure	1.4%	1.4%	2.9%	10.0%	84.3%
	Practices Festive Leisure	0.0%	0.0%	1.4%	5.6%	93.0%
